# Glycopeptidolipids, a Double-Edged Sword of the *Mycobacterium abscessus* Complex

**DOI:** 10.3389/fmicb.2018.01145

**Published:** 2018-06-05

**Authors:** Ana Victoria Gutiérrez, Albertus Viljoen, Eric Ghigo, Jean-Louis Herrmann, Laurent Kremer

**Affiliations:** ^1^Centre National de la Recherche Scientifique, Institut de Recherche en Infectiologie de Montpellier, UMR 9004, Université de Montpellier, Montpellier, France; ^2^CNRS, IRD 198, INSERM U1095, APHM, Institut Hospitalo-Universitaire Méditerranée Infection, UMR 7278, Aix-Marseille Université, Marseille, France; ^3^CNRS, Campus Joseph Aiguier, Marseille, France; ^4^2I, UVSQ, INSERM UMR 1173, Université Paris-Saclay, Versailles, France; ^5^INSERM, IRIM, Montpellier, France

**Keywords:** *Mycobacterium abscessus*, glycopeptidolipid, cell wall, pathogenesis, host/pathogen interactions

## Abstract

*Mycobacterium abscessus* is a rapidly-growing species causing a diverse panel of clinical manifestations, ranging from cutaneous infections to severe respiratory disease. Its unique cell wall, contributing largely to drug resistance and to pathogenicity, comprises a vast panoply of complex lipids, among which the glycopeptidolipids (GPLs) have been the focus of intense research. These lipids fulfill various important functions, from sliding motility or biofilm formation to interaction with host cells and intramacrophage trafficking. Being highly immunogenic, the induction of a strong humoral response is likely to select for rough low-GPL producers. These, in contrast to the smooth high-GPL producers, display aggregative properties, which strongly impacts upon intracellular survival. A propensity to grow as extracellular cords allows these low-GPL producing bacilli to escape the innate immune defenses. Transitioning from high-GPL to low-GPL producers implicates mutations within genes involved in biosynthesis or transport of GPL. This leads to induction of an intense pro-inflammatory response and robust and lethal infections in animal models, explaining the presence of rough isolates in patients with decreased pulmonary functions. Herein, we will discuss how, thanks to the generation of defined GPL mutants and the development of appropriate cellular and animal models to study pathogenesis, GPL contribute to *M. abscessus* biology and physiopathology.

## Introduction

*Mycobacterium abscessus* is a fast-growing non-tuberculous mycobacterium (NTM) and an emerging human pathogen that causes nosocomial skin and soft tissue infections (Brown-Elliott et al., 2012) but also pulmonary infections, especially in patients with cystic fibrosis (CF) and other lung disorders (Sermet-Gaudelus et al., 2003; Esther et al., 2010). Recent investigations reported mechanisms of virulence and physiopathological processes characterizing *M. abscessus* infection thanks to (i) genetic tools that allowed generation of defined mutants and transposon libraries, particularly useful to seek out genetic determinants of intracellular survival (Medjahed and Reyrat, 2009; Cortes et al., 2011; Gregoire et al., 2017; Laencina et al., 2018) and (ii) the development of various complementary cellular and animal models, which have allowed delineation of the early stages of the infection and the role of important cell types participating in controlling the infection and/or in the formation of granulomas (Ordway et al., 2008; Bernut et al., 2014a, 2017; Laencina et al., 2018). Evidence exists that granulomas harbor persistent *M. abscessus* for extended periods of time (Tomashefski et al., 1996; Medjahed et al., 2010). Additionally, these models have been used successfully to test the *in vivo* therapeutic efficacy of compounds against *M. abscessus*, considered as one of the most drug-resistant mycobacterial species (Bernut et al., 2014b; Dubée et al., 2015; Obregón-Henao et al., 2015; Dupont et al., 2016).

Like other NTMs, *M. abscessus* displays smooth (S) or rough (R) colony morphotypes, associated with distinct *in vitro* and *in vivo* phenotypes. This colony-based distinction is dependent on the presence (in S) or absence (in R) of surface-associated glycopeptidolipids (GPLs) (Howard et al., 2006; Medjahed et al., 2010). The presence or lack of GPL considerably influences important physiological and physiopathological aspects, including sliding motility or biofilm formation, interaction with host cells, intracellular trafficking in macrophages and virulence, ultimately conditioning the clinical outcome of the infection. This review gathers some of the most recent findings related to biosynthesis and transport of GPL in *M. abscessus*, the mechanisms driving the S-to-R switch and how this transition influences the surface properties of the bacilli, interaction with host cells, virulence and potentially the mode of transmission of *M. abscessus*.

## Genomics and Structural Aspects of GPL in *M. abscessus*

**FIGURE 1:**
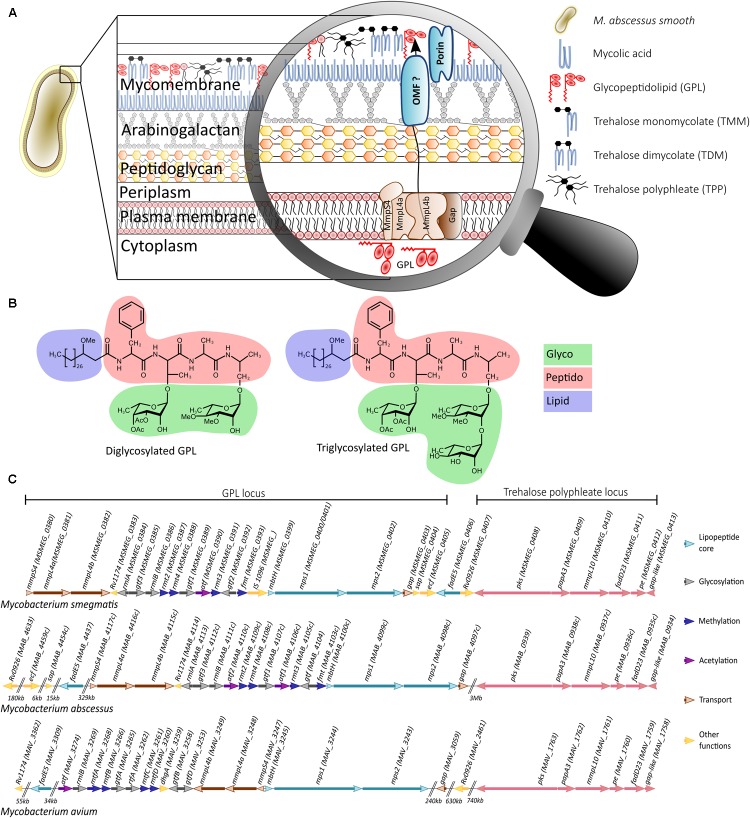
Cell wall localization, structure, and genomics of GPL. **(A)** Schematic representation of the *Mycobacterium abscessus* envelope, with a special focus on the plasma membrane proteins participating in the transport of GPL and on the inner and outer leaflets of the mycomembrane impregnated with various extractible lipids such as GPL. **(B)** Structure of the diglycosylated (apolar) and triglycosylated (polar) GPL. As GPL represent a highly heterogenous population of lipids, only one structure is depicted. Modifications can occur in the lipid chain length or in the hydroxylation/*O*-methylation status of the various monosaccharides. **(C)** Genomic organization of the *gpl* and *tpp* loci in *Mycobacterium smegmatis*, *Mycobacterium abscessus*, and *Mycobacterium avium*. Arrows indicating the transcription orientation of the different genes are drawn to scale. A color code has been used to specify the participation of these genes in synthesis of the lipopeptide core, glycosylation, methylation, acetylation, transport and other biological functions, as indicated. Genes in the *tpp* locus are displayed in pink.

The mycobacterial envelope comprises three layers: a typical plasma membrane, a complex cell wall partly resembling a Gram-positive wall and an outer layer (Daffé and Draper, 1998). Particularly unusual, the cell wall consists of a thick peptidoglycan layer covalently-linked to arabinogalactan, itself esterified by mycolic acids, forming the inner leaflet of the mycomembrane. In addition, a large variety of extractible lipids form the outer leaflet of the mycomembrane. Among these are the GPL, found in many NTM (**Figure 1A**). GPL are subdivided into alkali-stable C-type GPL and alkali-labile serine-containing GPL. The C-type GPL are found in saprophytic mycobacteria such as *Mycobacterium smegmatis* or in opportunistic pathogens like *Mycobacterium avium*, *Mycobacterium chelonae*, or *Mycobacterium abscessus* (Schorey and Sweet, 2008), whereas the alkali-labile serine-containing GPL were found in *Mycobacterium xenopi* (Besra et al., 1993). C-type GPL share a common lipopeptidyl core consisting of a mixture of 3-hydroxy and 3-methoxy C28-30 fatty acids amidated by a tripeptide-amino-alcohol core of D-Phe-D-*allo*-Thr-D-Ala-L-alaninol. This lipopeptide core is glycosylated with the *allo*-Thr linked to a 6-deoxy-α-L-talose and the alaninol linked to an α-L-rhamnose. These di-glycosylated GPL make up the less polar species (**Figure 1A**). In the case of *M. avium*, the 6-deoxytalose is non-methylated or 3-*O*-methylated, and the rhamnose is either 3-*O*-methylated or 3,4-di-*O*-methylated. *M. avium* GPL can also be *O*-acetylated at various locations, depending on the strain. In contrast, *M. smegmatis*, *M. chelonae*, and *M. abscessus* produce di-glycosylated GPL that contain a 3,4-di-*O*-acetylated 6-deoxytalose and a 3,4-di-*O*-methylated or 2,3,4-tri-*O*-methylated rhamnose (Villeneuve et al., 2003; Ripoll et al., 2007; Whang et al., 2017). These species also produce more polar GPL by the addition of a 2,3,4-tri-hydroxylated rhamnose to the alaninol-linked 3,4-di-*O*-methyl rhamnose. Although being structurally identical, triglycosylated GPL are more abundant in *M. abscessus* than in *M. smegmatis* (Ripoll et al., 2007). GPL are heterogenous in structure and vary according to the fatty acyl chain length and the degree of hydroxylation or *O*-methylation of the glycosidic moieties (**Figure 1B**).

The *gpl* locus is highly conserved in *M. smegmatis*, *M. abscessus*, and *M. avium* (**Figure 1C**) but differences exist, like the presence of an IS1096 in *M. smegmatis.* The tripeptide-aminoalcohol moiety of GPL is assembled by the products of *mps1* and *mps2* (Billman-Jacobe et al., 1999). The genes *gtf1* and *gtf2* catalyze glycosylation of the lipopeptide core whereas *gtf3* adds the extra rhamnose defining triglycosylated GPL. The genes *rmt2*, *rmt3*, and *rmt4* participate in *O*-methylation of the rhamnose and *fmt*, absent in *M. avium*, in *O*-methylation of the lipid moiety. In contrast to *M. smegmatis* which possesses a single *atf* gene involved in acetylation of the two positions of the deoxytalose, two genes, *atf1* and *atf2*, transfer the acetyl residues in a sequential manner in *M. abscessus* (Ripoll et al., 2007). Separated by *gtf3*, *rmlA* and *rmlB* are responsible for monosaccharide activation and epimerization. On the proximal end of the *gpl* locus is found *mmpS4*, *mmpL4a* and *mmpL4b* in an operon and encoding membrane proteins required for the transport of GPL across the plasma membrane (Medjahed and Reyrat, 2009; Deshayes et al., 2010; Bernut et al., 2016b). MmpS4 has been proposed to mediate formation of the GPL biosynthesis/transport machinery megacomplex located at the bacterial pole (Deshayes et al., 2010). GPL transport requires also the integral membrane protein Gap in *M. smegmatis* (Sondén et al., 2005) (**Figure 1A**). How GPL are translocated from the periplasmic space to the outer membrane, however, remains unknown. Additionally, a block of eight genes [*MSMEG_0406* (*fadE5*) to *MSMEG_0413* (*gap-like*)] in *M. smegmatis*, originally proposed to catalyze the lipid synthesis and attachment to the tripeptide-aminoalcohol moiety of GPL (Ripoll et al., 2007) was recently reattributed to the synthesis of trehalose polyphleates (TPP) (Burbaud et al., 2016). In *M. abscessus*, this cluster is far away from the main *gpl* locus with *Rv0926* and *fadE5* being scattered in the *M. abscessus* chromosome (**Figure 1C**).

## Molecular Mechanisms of the Smooth-To-Rough Transition and Associated Phenotypes

**FIGURE 2:**
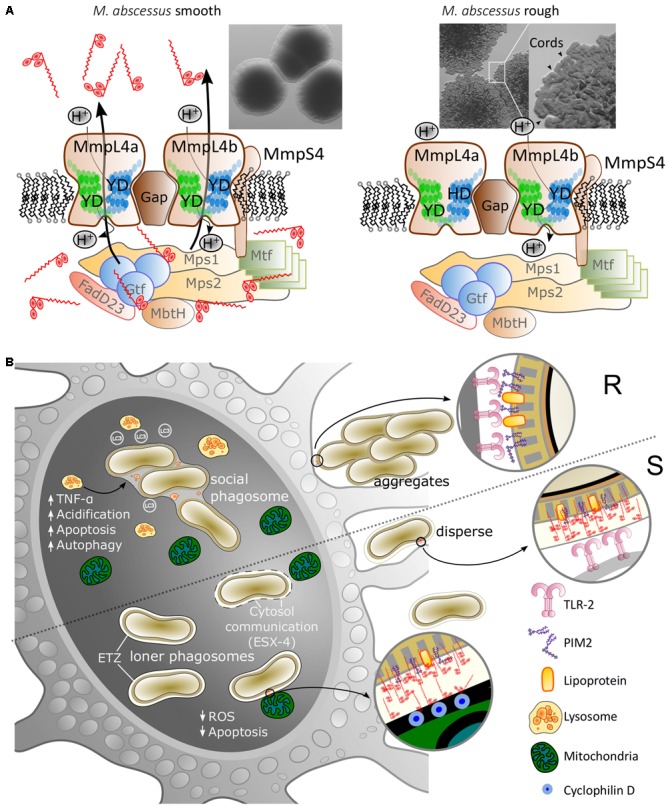
Glycopeptidolipid (GPL) composition influences the colonial morphology and host cell interactions of *M. abscessus*. **(A)** Morphology of the high-GPL producing smooth (S) morphotype (left panel) and low-GPL producing rough (R) morphotype (right panel). The production and transport of GPL requires multiple cytosolic biosynthetic enzymes and transmembrane proteins. Mutations in genes involved in GPL production/transport occurring *in vivo* or introduction of point mutations of crucial Tyr/Asp (Y/D) couples in MmpL4a/MmpL4b participating in the proton relay lead to the arrest of lipid transport and a rough morphotype (right panel). R strains are typified by the production of serpentine cords (inset, arrowheads). **(B)** Inhibition of the macrophage apoptotic response by GPL covering *M. abscessus* S occurs through mitochondrial targeting and interaction with cyclophilin D, reduced production of radical oxygen species (ROS) and limitation of the spread of *M. abscessus* among macrophages. In contrast, the loss of GPL in *M. abscessus* R uncovers immunostimulatory TLR-2 agonists, such as PIM_2_ and lipoproteins, leading to an exacerbated pro-inflammatory response that contributes to acute and severe infections.

Comparative genomics to understand the molecular basis of the S and R phenotypes using isogenic S and R pairs revealed multiple indels or single nucleotide polymorphisms within the *gpl* locus (Pawlik et al., 2013). A single nucleotide deletion in *mmpL4b* and nucleotide insertions in *mps1* were identified in the R variants when compared to the S variants from the three different isogenic S/R couples. Moreover, RNA sequencing demonstrated that S and R isogenic strains differed considerably at the transcriptomic level, with the transcriptional extinction of *mps1*, *mps2*, and *gap* in the R strain caused by an insertion in the 5′-end of *mps1* (Pawlik et al., 2013). Additional mutations in *mps2*, *mmpL4a*, and *mmpS4* were subsequently identified in R strains isolated from later disease stages (Park et al., 2015). Disruption of *mmpL4b* in *M. abscessus* S was initially reported to abrogate GPL production, leading to a rough colonial morphotype (Medjahed and Reyrat, 2009; Nessar et al., 2011) (**Figure 2A**). Point mutations in MmpL4a at Tyr842 or MmpL4b at Tyr854, corresponding to two critical residues presumably involved in the proton-motive force of the MmpL proteins, were also associated with loss of GPL production (Bernut et al., 2016b) (**Figure 2A**), suggesting that no functional redundancy exists between MmpL4a and MmpL4b.

Hydrophilic and hydrophobic properties of bacteria can influence surface adhesion and biofilm formation (Krasowska and Sigler, 2014). As shown in *M. smegmatis* (Recht and Kolter, 2001), *M. abscessus* (Howard et al., 2006) and *M. bolletii* (Bernut et al., 2016b) the presence of GPL in the S variants facilitates sliding across the surface of motility agar and biofilm formation on the liquid medium/air interface whereas lack of GPL promotes bacterial aggregation (Brambilla et al., 2016) and cording (Howard et al., 2006; Bernut et al., 2014a, 2016b). However, whether *M. abscessus* R fails at producing biofilms was recently readdressed and proposed that it can grow in biofilm-like structures, which, like S biofilms, are significantly more tolerant than planktonic cultures to acidic pH, hydrogen peroxide, and drugs (Clary et al., 2018). In *M. smegmatis*, the nucleoid-associated protein Lsr2 negatively regulates GPL production (Kocíncová et al., 2008) and plays a role during the initial stages of biofilm development (Yang et al., 2017). Despite the presence of an *lsr2* gene in *M. abscessus*, which is up-regulated in the R variant (Pawlik et al., 2013), the contribution of Lsr2 in regulating GPL expression, sliding motility and biofilm formation remains to be established.

External factors, such as sub-inhibitory antibiotic concentrations, can promote a transient S-to-R change with more aggregated cultures and a higher resistance to phagocytosis (Tsai et al., 2015). Strangely, these phenotypes were neither linked to a loss of GPL production nor to the differential expression of genes within the *gpl* cluster, but were rather mediated by *MAB_3508c*, homologous to *whiB7* and conferring extreme resistance to antibiotics in *M. abscessus* (Hurst-Hess et al., 2017). In contrast, another study reported that sub-inhibitory amikacin treatment, also leading to a S-to-R transition, was associated with decreased GPL, resulting from down-regulation of several *gpl* biosynthetic genes (Lee et al., 2017). Overall, these results suggest that exposure to sub-inhibitory amikacin doses may induce alterations in GPL content, increase virulence and influence the outcome of the infection as well as the therapeutic efficacy of drugs.

## Presence or Loss of GPL Conditions Bacterial Surface Properties and Interactions With Host Cells

The S and R variants interact differently with host cells and exhibit different intracellular behaviors, as first reported in human monocytes with the R variant persisting longer in these cells (Byrd and Lyons, 1999) and then confirmed in other phagocytes (Howard et al., 2006; Roux et al., 2016). Macrophages encountering the aggregative R strain, are incapable of engulfing clumps of R bacteria, which remain embedded in phagocytic cups on the exterior of the cells (Roux et al., 2016). However, smaller clumps are phagocytosed, resulting in social phagosomes (containing numerous bacilli) that rapidly fuse with lysosomes. In addition, R variant-containing THP1 cells were more acidified, more autophagic and more apoptotic than those infected with the S variant. In contrast, in the majority of S variant-containing phagosomes, a continuous tight apposition is maintained between the phagosome membrane and the mycobacterial cell envelope, leading to phagosome maturation blockage and the absence of acidification in S-infected macrophages. Another conspicuous trait of the S-containing phagosomes is the occurrence of a large electron translucent zone (ETZ) enclosing the bacilli (**Figure 2B**). This ETZ is barely detected in R-containing phagosomes or in phagosomes containing S strains mutated in either *mmpL4a* or *mmpL4b*, indicating that the ETZ relies on the presence of cell surface GPL (Bernut et al., 2016b; Roux et al., 2016). Interestingly, infection with *M. abscessus* S, but not R, leads to phagosome membrane lesions, suggesting that, similarly to pathogenic slow-growing mycobacteria, this variant has the capacity to induce phagosome–cytosol communications (Simeone et al., 2012), through a mechanism that likely involves the type VII secretion system ESX-4 (Laencina et al., 2018).

The distinct mechanisms responsible for the S- and R-induced responses in macrophages are being delineated, highlighting a model whereby the loss of GPL at the surface unmasks underlying phosphatidyl-*myo*-inositol dimannoside (PIM_2_) (Rhoades et al., 2009) and lipoproteins (Roux et al., 2011). These TLR-2 agonists stimulate the expression of TNF and intense inflammation. Exacerbation of this response can also lead to tissue lesions associated with R strains. In S strains, by covering underlying immunostimulatory cell wall components, GPL may delay the activation of the immune response during early infection stages and facilitate colonization by preventing TLR-2 signaling in the respiratory epithelial cells (Davidson et al., 2011).

Apoptosis represents an innate response of cells to restrict multiplication of intracellular pathogens (Lamkanfi and Dixit, 2010). *M. abscessus* R was found to be more apoptotic than *M. abscessus* S in different types of macrophages (Roux et al., 2016; Whang et al., 2017). Supporting these findings, purified GPL from *M. abscessus* S inhibits macrophage apoptosis, presumably by suppressing the production of radical oxygen species (ROS), the release of cytochrome c and by preserving the mitochondrial transmembrane potential (Whang et al., 2017) (**Figure 2B**). This mechanism appears to be mediated by targeting of acetylated GPL to the mitochondria where they interact with cyclophilin D, a component of the mitochondrial permeability transition pore (MPTP), inhibiting the MPTP that results in a block on cell death in similar fashion to cyclosporin A. That exogenous GPL-dependent apoptosis inhibition restricts intracellular growth and spreading of *M. abscessus* R, suggests also that GPL may limit *M. abscesssus* virulence (Whang et al., 2017).

Collectively, these observations emphasize the diversity of the infection programs orchestrated by S and R variants. While the S variant is promptly phagocytosed by macrophages without immediately affecting cell survival, phagocytosis of the R variant is more harmful to macrophage viability and, following apoptosis, the released bacteria replicate extracellularly in the form of serpentine cords (**Figure 2A**).

## Impact of the GPL Content on Virulence

Epidemiological surveys document the prominence of the R strain in patients with severe pulmonary infections (Catherinot et al., 2009) and with chronic colonization of the airways in CF patients (Jönsson et al., 2007), but the exact proportions of S and R forms in these populations remain largely unknown. This distinction is, however, of crucial importance considering that the cord-forming R variant causes much more aggressive and invasive pulmonary disease that ends in severe respiratory failure. Further alarm is raised by several studies using various cellular and animal models, confirming the increased virulence of the R over the S form (Bernut et al., 2017). Among these models, the zebrafish (*Danio rerio*) has been proposed as a relevant and genetically tractable host–pathogen conjugate for dissecting *M. abscessus* interactions with host cells (Bernut et al., 2015). This led to important breakthroughs regarding mechanisms of *M. abscessus* pathogenesis, involving cording and granuloma formation (Bernut et al., 2014a) or the importance of the TNF response in controlling the infection and establishment of protective granulomas (Bernut et al., 2016a). S-to-R transitioning is associated with exacerbation of the bacterial burden, the formation of massive serpentine cords, abscess formation, notably in the central nervous system, and increased larval killing (Bernut et al., 2014a). Deletion of *MAB_4780*, encoding a dehydratase required for cording, resulted in extreme attenuation in wild-type and immunocompromized larvae (Halloum et al., 2016), further incriminating cording as a major virulence determinant in strains lacking GPL. Replacing the endogenous *mmpS4-mmpL4a-mmpL4b* promoter with the leaky acetamidase promoter from *M. smegmatis* in *M. abscessus* S resulted in a strain with low-GPL levels, but still aggregating in culture with a rough appearance, similar to R strains. In zebrafish, this mutant exhibited an intermediate virulence phenotype with a delay in killing compared to the R strain. Moreover, the number and size of the abscesses in larvae infected with this low-GPL producing strain were significantly reduced compared to the R strain (Viljoen et al., 2018). This indicates that low-GPL levels impede the induction of the physiopathological signs and virulence of *M. abscessus* R, confirming the opposite relationship between the amount of GPL and virulence. In addition, the S variant is more hydrophilic than the R variant or the rough low-GPL producing strain. This suggests that lack of the hydrophilic GPL components is responsible for their increased hydrophobicity over the S strain and confirm a positive correlation between GPL production and hydrophilicity.

Supporting the theory of transmission *via* aerosols from the environment, *M. abscessus*, along with other NTM, was isolated from household water and shower aerosols in the homes of patients with pulmonary disease (Thomson et al., 2013). Importantly, a direct person-to-person transmission of *M. abscessus* by aerosol inhalation has been asserted in recent world-wide surveys, although the link between morphology/GPL profile and mode of transmission remains to be investigated (Bryant et al., 2013, 2016).

## Conclusion and Perspectives

Fundamental aspects of the *M. abscessus* lifecycle rely on the beneficial effects of GPL in promoting and facilitating the early stages of colonization of the S variant, presumably the major form existing in the environment. By covering the bacilli, the highly immunogenic GPL induce a strong humoral response in infected individuals and it is possible that this strong immune pressure leads to selection of GPL-deficient strains, allowing *M. abscessus* to escape the anti-GPL response and the emergence of R bacilli. The lack of GPL, in turn, leads to increased apoptosis, promoting extracellular replication and cording, acute infection and the most severe forms of the disease. This has also detrimental consequences for the host since, by unmasking other pro-inflammatory cell-surface components, the loss of GPL translates to severe inflammation and lung damage. Therefore, the double edged sword effect of GPL allows *M. abscessus* to efficiently transition between a colonizing environmental micro-organism to an invasive human pathogen. Given the importance of the GPL content in driving the interaction with host cells and in conditioning the issue of the infection, it appears important to pay more attention to the variant (S or R) selected for experimental infections and to systematically report the morphotype of the strains isolated in clinical studies.

Important unsolved questions remain on GPL in *M. abscessus*. Future investigations should describe the complete GPL export machinery since, while transfer of GPL across the plasma membrane has been addressed to some extent, additional unidentified outer-membrane proteins are likely to participate in this important physiological process. So far, the literature only reports the effects of near total loss of GPL in *M. abscessus*, portraying an incomplete picture of the functions of these lipids in the physiology of this pathogen. Polar GPL species in *M. smegmatis* are only produced under carbon starvation and induce smooth-colony formation (Ojha et al., 2002) opening up the possibility that in *M. abscessus* GPL composition is modulated in response to changing environments. Therefore, studies are required to address whether GPL composition affects *M. abscessus* persistence and/or host inflammation, as well as how the dynamics of GPL production, potentially mediated by yet unidentified factors, influences adaptation of *M. abscessus* to its host.

## Author Contributions

AG designed the figures. All authors contributed to writing the manuscript.

## Conflict of Interest Statement

The authors declare that the research was conducted in the absence of any commercial or financial relationships that could be construed as a potential conflict of interest.
